# Assessment of Liver Function for Evaluation of Short- and Long-Term Outcomes in Type B Aortic Dissection Patients Undergoing Thoracic Endovascular Aortic Repair

**DOI:** 10.3389/fcvm.2021.643127

**Published:** 2021-05-12

**Authors:** Jitao Liu, Min Wu, Enmin Xie, Lyufan Chen, Sheng Su, Hongke Zeng, Qingshan Geng, Fan Yang, Jianfang Luo

**Affiliations:** ^1^Department of Cardiology, Guangdong Provincial Key Laboratory of Coronary Heart Disease Prevention, Guangdong Cardiovascular Institute, Guangdong Provincial People's Hospital, Guangdong Academy of Medical Sciences, Guangzhou, China; ^2^Department of Cardiac Surgery, Guangdong Cardiovascular Institute, Guangdong Provincial People's Hospital, Guangdong Academy of Medical Sciences, Guangzhou, China; ^3^The Second School of Clinical Medicine, Southern Medical University, Guangzhou, China; ^4^School of Medicine, South China University of Technology, Guangzhou, China; ^5^Department of Emergency and Critical Care Medicine, Guangdong Provincial People's Hospital, Guangdong Academy of Medical Sciences, Guangzhou, China

**Keywords:** type B aortic dissection, thoracic endovascular aortic repair, liver function, mortality, aspartate transaminase to platelet ratio index, model of end-stage liver disease, albumin-bilirubin score

## Abstract

**Background and Aims:** Patients with decreased liver function suffer from poor outcomes when undergoing procedures. We aimed to explore the impact of liver fibrosis identified by aspartate transaminase-to-platelet ratio index (APRI) and poor liver functional reserve assessed by a model of end-stage liver disease (MELD) and albumin–bilirubin(ALBI) score on the prognosis of patients with type B aortic dissection (TBAD) undergoing thoracic endovascular aortic repair (TEVAR).

**Methods:** A retrospective analysis of a prospectively maintained database from 2010 to 2017 was performed. APRI > 0.5 was used to identify those with significant liver fibrosis. Logistic and Cox regression analyses were performed to investigate the association between liver fibrosis, MELD, and ALBI with adverse events.

**Results:** TEVAR was performed on 812 TBAD patients including 35 with liver fibrosis and 777 without. Twenty-four (3.0%) patients deceased during hospitalization and 69 (8.8%) patients died after a median 48.2 months follow-up. Multivariable analysis revealed that liver fibrosis, MELD, and ALBI were independently associated with in-hospital [fibrosis: odds ratio (OR) 23.73, 95% confidence interval (CI) 8.89–63.33, *P* < 0.001; MELD: OR 1.08, 95% CI 1.03–1.14, *P* = 0.003; ALBI: OR 4.45; 95% CI 1.56–12.67, *P* = 0.005] and follow-up mortality [fibrosis: hazard ratio (HR) 4.69, 95% CI 1.93–11.42, *P* = 0.001; MELD: HR 1.07, 95% CI 1.04–1.10, *P* < 0.001; ALBI: HR 2.88, 95% CI 1.53–5.43, *P* = 0.001]. The association was further corroborated by a subgroup analysis.

**Conclusion:** Liver fibrosis and poor liver functional reserve could significantly increase the morbidity and mortality after TEVAR. APRI, MELD, and ALBI should be calculated and routinely used for preoperative risk stratification. Strict preoperative preparation and elaborate postoperative care are necessary to improve these patients' prognosis.

## Introduction

Type B aortic dissection (TBAD) is a catastrophic cardiovascular disease with high morbidity and mortality (1). Thoracic endovascular aortic repair (TEVAR) is considered as a therapeutic approach for TBAD with promising results. However, the early and late postoperative mortality remain high, especially for patients with multiple comorbidities (2). For early identification of patients at high risk, an accurate prognostic evaluation is critical for physicians and patients.

Poor liver function has been demonstrated to be an important prognostic risk factor for patients undergoing cardio-thoracic surgery (3–6). Notably, cardiovascular surgery is associated with greater mortality in patients with liver dysfunction than most other surgical procedures (7). Some researchers considered that the outcomes were acceptable to perform endovascular procedures in patients with liver disease, while others thought that patients with extremely poor liver function might not be suitable for endovascular therapy due to an increased risk of severe coagulation disorders (4–6). Therefore, a careful evaluation of the degree of preoperative liver function in order to stratify patients before TEVAR may optimize patient management and improve prognosis. However, research examining the impact of liver fibrosis and decreased liver functional reserve in patients with TBAD undergoing TEVAR is scarce.

The model of end-stage liver disease (MELD) and the aspartate transaminase–platelet ratio index (APRI) are recommended by the World Health Organization (WHO) as effective tools to evaluate the presence and severity of liver disease (8, 9). Recently, the albumin–bilirubin (ALBI) score, which is introduced to assess liver functional reserve in cirrhosis and hepatocellular carcinoma, showed appropriate or better capacity to evaluate liver functional reserve than the Child–Pugh classification does (10). In addition, MELD and ALBI reflect dynamic changes in liver function, which more correspond to the fact that the degree of liver functional reserve in patients requiring intervention might vary from mildly deteriorated liver function to late stage of hepatic damage (11, 12). Nevertheless, these widely used prognostic scoring systems have not been applied for patients with TBAD undergoing TEVAR.

Thus, we hypothesize that liver fibrosis and liver functional reserve could indicate the prognosis of TBAD patients undergoing TEVAR. Consequently, the goal of this study is to evaluate the impact of liver fibrosis identified by APRI and liver functional reserve assessed by MELD and ALBI in patients with TBAD undergoing TEVAR.

## Methods

### Study Population

**Figure 1:**
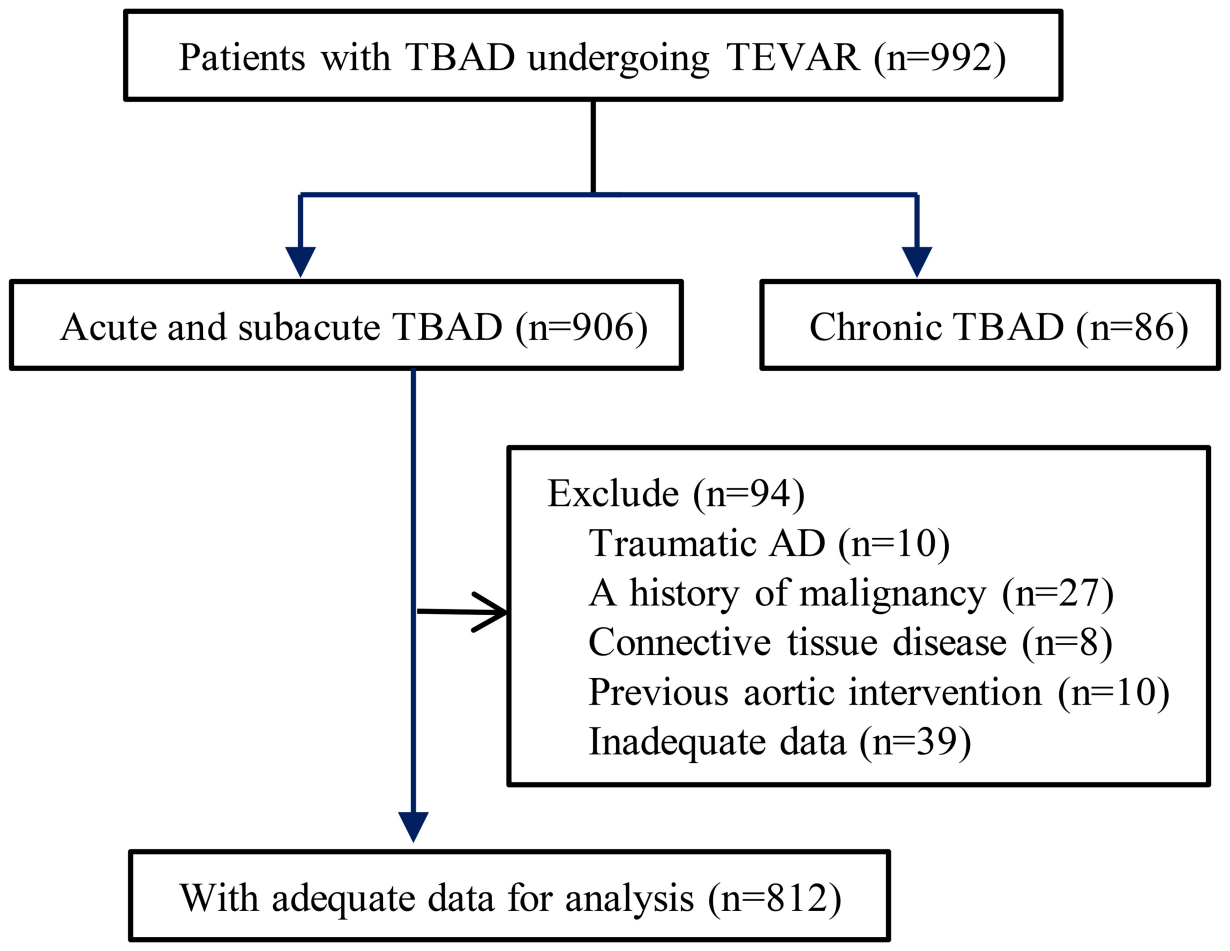
Flow chart demonstrating the inclusion of patients in the study. TBAD, type B aortic dissection; TEVAR, thoracic endovascular aortic repair.

Between January 2010 and December 2017, all patients with acute or subacute TBAD who underwent TEVAR procedures at our hospital were eligible for inclusion in this analysis. Patients meeting the following criteria were excluded: (1) blunt traumatic thoracic aortic injury, (2) malignant tumor, (3) connective tissue disease, (4) previous aortic intervention, and (5) inadequate data (Figure 1). All patients received computed tomography angiography (CTA) and were evaluated by an interdisciplinary board composed of cardiologists, endovascular surgeons, cardiovascular surgeons, and radiologists. The maximum outer-to-outer diameter of the aorta, the extent of the dissection, and false lumen status were evaluated by a dedicated Aquarius iNtuition software (TeraRecon, San Mateo, CA, USA). This study was approved by the ethics committee of Guangdong Provincial People's Hospital (#201807). There was no need for informed consent due to the retrospective nature of the analysis.

### Procedures

Indications for TEVAR in patients with complicated TBAD were consistent with the European Society of Cardiology (ESC) 2014 guidelines (1). Patients with uncomplicated TBAD underwent TEVAR when the true lumen was severely compressed or the false lumen was >22 mm (13). The details of the procedure at our hospital have been previously described (13). Briefly, the procedures were performed with appropriate anatomy in a cardiac catheterization room. All stent grafts were deployed retrogradely via a percutaneous femoral artery access to obliterate the proximal entry tear. The left subclavian arteries were covered when necessary to obtain 1.5–2 cm proximal landing zone. The choice of reconstruction of the left subclavian artery mainly depends on the vertebrobasilar circulation, assessed by the operators. The diameters of aortic stent grafts were generally oversized by 5–10% according to the aortic pathologies. Most patients were treated with one stent, unless the initial graft did not cover the entry tear and the expansion of the true lumen. Balloon angioplasty of the proximal seal zone was avoided if possible to prevent retrograde extension of the dissection into the aortic arch.

### Definition

Acute TBAD was defined as a type B aortic dissection occurring <14 days after the onset of symptoms, whereas subacute TBAD was defined as a dissection with an elapsed time between 15 and 90 days (1). Complicated TBAD was described as persistent or recurrent pain, uncontrolled hypertension despite full medication, early aortic expansion, malperfusion, and signs of rupture (hemothorax and increasing periaortic and mediastinal hematoma) (1).

Venous blood was collected from all patients after admission. Blood samples were drawn into tubes containing 3.2% sodium citrate and sent to the laboratory for testing as soon as possible. The APRI was calculated from dividing the aspartate transaminase (AST) level by platelet level, where AST was in units per liter and platelet was in 10^9^ per liter (6). The ALBI was calculated by the formula (log_10_ bilirubin × 0.66) – (albumin × 0.085), where bilirubin was in moles per liter and albumin was in grams per liter (10). The MELD score was calculated using the United Network for Organ Sharing modifications as follows: MELD = 9.57 × ln (serum creatinine) + 3.78 × ln (serum bilirubin) + 11.20 × ln (INR) + 6.43, where serum creatinine and serum bilirubin are in milligrams per deciliter and INR represents international normalized ratio (8).

The primary outcome was in-hospital death and follow-up all-cause mortality. In-hospital major adverse clinical events (MACE), including cerebral infarction, limb or visceral ischemia, spinal cord ischemia, secondary intervention, or death, were considered as the secondary outcome.

### Follow-Up Protocol

All in-hospital survival patients underwent clinical and imaging follow-up at 3, 6, and 12 months and annually thereafter. Each patient was suggested to return to the hospital for a review at corresponding intervals. Data including symptoms, medication, imaging findings, and other relevant conditions were collected during follow-up. Assessment of patients' condition was mainly completed by an outpatient clinic visit or telephone interview.

### Statistical Analysis

Descriptive statistics are presented as the mean ± standard deviation or median with interquartile range for continuous variables and as numbers with frequencies for categoric variables. D-dimer level was log-transformed to account for the skewed distribution. Continuous data was compared using Student's *t*-test of normal distribution or the Mann–Whitney *U*-test for non-normal distribution. Categorical data was compared using the chi-square test or Fisher's exact test between groups, and then multiple comparisons were performed with Bonferroni corrections.

Univariate and multivariate logistic regression analyses and Cox survival models were carried out to explore the relationship of variables with in-hospital mortality and MACE, as well as follow-up mortality, respectively. Factors in the univariate analysis with a *P* < 0.1 were entered into the multivariate regression model to identify independent predictors by a stepwise forward likelihood ratio method. A subgroup analysis performed by disease pathology (acute or subacute) and severity (uncomplicated or complicated) was undertaken to investigate the consistency of the conclusion among different sub-populations. A threshold of 0.5 of APRI was used to indicate significant liver fibrosis (6). X-tile program was applied for determining optimal threshold of ALBI with regard to total death during follow-up (14), and a value of −1.58 was then generated for prognostic discrimination. All statistical analyses were performed using R software (version 3.5.1). A value of *P* < 0.05 was considered significant.

## Results

### Clinical Characteristics

A total of 812 TBAD patients undergoing TEVAR were analyzed, including 35 (4.3%) with liver fibrosis and 777 (95.7%) without. The MELD score [fibrosis: 14.02 (10.08–19.34) vs. non-fibrosis: 8.73 (7.29–10.90); *P* < 0.001] and ALBI score [fibrosis: −1.84 (−1.92 to −1.36) vs. non-fibrosis: −1.97 (−2.26 to −1.70); *P* < 0.001] of patients with liver fibrosis were significantly higher than those without liver fibrosis. There were 543 (66.9%) patients with a MELD score <10, 188 (23.1%) patients with a MELD score between 10 and 15, and 81 (10.0%) patients with a MELD score >15. Based on the X-tile program, 138 (17.0%) patients were classified into high ALBI group (ALBI > −1.58) and 674 (83.0%) patients were classified into low ALBI group (ALBI ≤ −1.58).

Patients with liver fibrosis had a higher incidence of the involvement of abdominal arteries, higher D-dimer and estimated glomerular filtration rate level, as well as worse liver functional reserve (elevated alanine transaminase, AST, bilirubin, and INR and decreased albumin) (Table 1). There was no significant difference between patients with/without liver fibrosis concerning the demographic characteristics, co-existing medical conditions, and medications at admission (Table 1).

**Table 1:** Baseline characteristics of patients with and without liver fibrosis.

**Variables**	**No fibrosis (APRI ≤0.5; *n* = 777)**	**Fibrosis (APRI >0.5; *n* = 35)**	***P***
Age, years	54.0 (46.0–63.0)	52.0 (46.0–61.0)	0.403
Male	670 (86.2)	34 (97.1)	0.063
Stage			0.258
Acute	629 (81.0)	31 (88.6)	
Subacute	148 (19.0)	4 (11.4)	
Complicated	477 (61.4)	21 (60.0)	0.869
Hypertension	654 (84.2)	30 (85.7)	0.806
Coronary artery disease	118 (15.2)	5 (14.3)	0.884
Diabetes mellitus	51 (6.6)	2 (5.7)	0.842
Anemia	373 (48.0)	20 (57.1)	0.290
Stroke	23 (3.0)	3 (8.6)	0.065
Chronic kidney disease	84 (10.8)	8 (22.9)	0.028
Abdominal aortic aneurysm	25 (3.2)	0 (0)	0.281
Diameter, mm	37.1 (34.0–42.6)	38.0 (34.0–45.6)	0.588
Extent of the dissection			0.215
Confined in thoracic aorta	181 (23.3)	5 (14.3)	
Extended to abdominal aorta	596 (76.7)	30 (85.7)	
False lumen status			0.837
Patent	525 (67.6)	25 (71.4)	
Partially thrombosed	225 (29.0)	10 (28.6)	
Completely thrombosed	27 (3.5)	0 (0)	
The involvement of coeliac artery	223 (28.7)	16 (45.7)	0.031
The involvement of SMA	135 (17.4)	12 (34.3)	0.011
The involvement of renal artery			0.057
None	446 (57.4)	14 (40.0)	
Unilateral	301 (38.7)	18 (51.4)	
Bilateral	30 (3.9)	3 (8.6)	
Renal cysts	173 (22.3)	5 (14.3)	0.264
lg (D-dimer)	3.4 (2.9–3.6)	3.5 (3.3–3.9)	0.002
Platelets, 10^9^/L	208.3 (167.8–274.2)	130.7 (90.0–152.0)	<0.001
ALT, U/L	21.0 (15.0–34.0)	88.0 (62.0–164.0)	<0.001
AST, U/L	21.0 (17.0–29.0)	114.0 (78.0–188.4)	<0.001
eGFR, mL/min/1.73 m^2^	82.7 (59.8–97.0)	49.1 (24.0–86.8)	<0.001
Creatinine, mg/dL	0.99 (0.82–1.31)	1.64 (0.92–2.74)	<0.001
Albumin, g/L	33.0 (29.4–36.0)	30.7 (26.2–33.3)	0.006
ALP, U/L	71.0 (56.0–91.0)	75.0 (51.0–108.0)	0.604
GGT, U/L	37.0 (22.0–69.0)	60.0 (28.5–111.0)	0.087
Bilirubin, μmol/L	16.4 (11.4–22.7)	19.5 (14.1–26.6)	0.040
INR	1.06 (1.01–1.11)	1.15 (1.07–1.24)	<0.001
**Medications at admission**
Antiplatelet drugs	5 (14.3)	132 (17.0)	0.676
ACEI	4 (11.4)	159 (20.5)	0.192
ARB	13 (37.1)	355 (45.7)	0.320
Beta-blockers	31 (88.6)	728 (93.7)	0.230
Calcium channel blockers	24 (68.6)	594 (76.4)	0.285

*SMA, superior mesenteric artery; ALT, alanine aminotransferase; AST, aspartate aminotransferase; eGFR, estimated glomerular filtration rate; ALP, alkaline phosphatase; GGT, gamma-glutamyl transferase; INR, international normalized ratio; ACEI, angiotensin-converting enzyme inhibitors; ARB, angiotensin receptor blockers*.

During the hospital stay, 24 (3.0%) patients deceased (detailed in the Supplementary Table 1), 22 (2.7%) patients had a cerebral infarction, 18 (2.2%) patients had a limb ischemia, 4 (0.5%) patients had a visceral ischemia, 11 (1.4%) patients had a spinal cord ischemia, and 10 (1.2%) patients required a secondary intervention. After a median 48.2 months (25.5–73.0) follow-up, 69 (8.8%) patients died (Table 2).

**Table 2:** Post-operative outcomes of patients with and without liver fibrosis.

**Variables**	**No fibrosis (APRI ≤0.5; *n* = 777)**	**Fibrosis (APRI >0.5; *n* = 35)**	***P***
**In-hospital events**
Mortality	14 (1.8)	10 (28.6)	<0.001
Cerebral infarction	20 (2.6)	2 (5.7)	0.244
Limb ischemia	15 (1.9)	3 (8.6)	0.038
Visceral ischemia	2 (0.3)	2 (5.7)	0.010
Spinal cord ischemia	9 (1.2)	2 (5.7)	0.078
Re-intervention	10 (1.3)	0 (0)	>0.999
Follow-up mortality	63 (8.3)	6 (24.0)	0.017

*APRI, Aspartate transaminase to platelet ratio index*.

### Liver Function Assessment With In-hospital Outcomes

**Figure 2:**
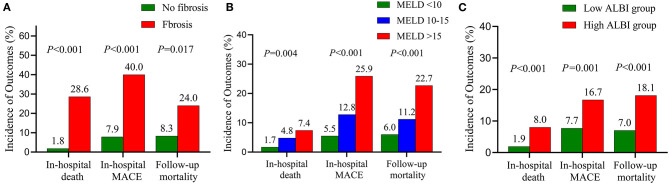
Prevalence of adverse events stratified by the fibrosis **(A)**, the MELD score **(B)** and the ALBI score **(C)**. MELD, model of end-stage liver disease; ALBI, albumin–bilirubin; MACE, major adverse clinical events.

When in-hospital outcomes were assessed, patients with liver fibrosis had significantly higher in-hospital death (28.6 vs. 1.8%; *P* < 0.001) and in-hospital MACE (40.0 vs. 7.9%; *P* < 0.001) (Figure 2). Liver fibrosis was demonstrated to be a significant risk factor for in-hospital death [odds ratio (OR), 23.73; 95% confidence interval (CI), 8.89–63.33; *P* < 0.001] and MACE (OR, 5.92; 95% CI, 2.75–12.77; *P* < 0.001) after adjusting for potential confounders (Table 3 and Supplementary Tables 2, 3).

**Table 3:** Results of multivariate regression analysis ^*^ for post-operative outcomes.

**Variables**	**APRI (>0.5 vs**. **≤0.5)**		**MELD**		**ALBI**	
	**OR/HR (95% CI)**	***P***	**OR/HR (95% CI)**	***P***	**OR/HR (95% CI)**	***P***
In-hospital death	23.73 (8.89–63.33)	<0.001	1.08 (1.03–1.14)	0.003	4.45 (1.56–12.67)	0.005
In-hospital MACE	5.92 (2.75–12.77)	<0.001	1.05 (1.002–1.11)	0.038	2.22 (1.19–4.15)	0.013
Follow-up mortality	4.69 (1.93–11.42)	0.001	1.07 (1.04–1.10)	<0.001	2.88 (1.53–5.43)	0.001

*APRI, Aspartate transaminase to platelet ratio index; MELD, model of end-stage liver disease; ALBI, albumin-bilirubin; MACE, major adverse clinical events*.

^*^Details in the Supplementary Tables 2–4.

We next conducted an analysis according to MELD and ALBI scores. In-hospital death (MELD <10, 1.7%; MELD 10–15, 4.8%; MELD > 15, 7.4%; *P* = 0.004) and in-hospital MACE (MELD < 10, 5.5%; MELD 10–15, 12.8%; MELD > 15, 25.9%; *P* < 0.001) differed across MELD categories with a stepwise increase noted (Figure 2). A similar trend was observed with increasing ALBI score (Figure 2). Multivariate analysis revealed that MELD and ALBI were also independent indicators for in-hospital death (MELD: OR 1.08, 95% CI 1.03–1.14, *P* = 0.003; ALBI: OR 4.45, 95% CI 1.56–12.67, *P* = 0.005) and MACE (MELD: OR 1.05, 95% CI 1.002–1.11, *P* = 0.038; ALBI: OR 2.22, 95% CI 1.19–4.15, *P* = 0.013) (Table 3 and Supplementary Tables 2, 3).

The ROC curve analysis was carried out to determine the predictive value for in-hospital outcomes. The AUC (95% CI) of APRI, MELD, and ALBI for in-hospital death were 0.71 (0.59–0.84), 0.66 (0.54–0.78), and 0.64 (0.52–0.76), respectively. The AUC (95% CI) of APRI, MELD, and ALBI for in-hospital MACE were 0.63 (0.55–0.70), 0.67 (0.60–0.74), and 0.62 (0.55–0.68), respectively.

### Liver Function Assessment With Follow-Up Mortality

Liver fibrosis compared with non-fibrosis was associated with higher follow-up mortality (24.0 vs. 8.3%; *P* = 0.017). Multivariate analysis revealed that liver fibrosis could profoundly affect the follow-up survival in TBAD patients undergoing TEVAR [hazard ratio (HR), 4.69; 95% CI, 1.93–11.42; *P* = 0.001] (Table 3 and Supplementary Table 4).

Similarly, increasing follow-up mortality was observed with the elevated MELD grades (MELD <10, 6.0%; MELD 10–15, 11.2%; MELD > 15, 22.7%; *P* < 0.001) and ALBI grades (ALBI ≤ −1.58, 7.0%; ALBI > −1.58, 18.1%; *P* < 0.001) (Figure 2). Multivariate analysis showed that MELD (HR, 1.07; 95% CI, 1.04–1.10; *P* < 0.001) and ALBI (HR, 2.88; 95% CI, 1.53–5.43; *P* = 0.001) were independent predictors for follow-up mortality (Table 3 and Supplementary Table 4).

In the subgroup analysis, we stratified the patients by age (<65 vs. ≥65 years), phase of disease (acute vs. subacute), severity of disease (complicated vs. uncomplicated), renal function (eGFR <60 vs. eGFR ≥60 ml/min/1.73 m^2^), coronary artery disease status (yes vs. no), and anemia status (yes vs. no). Multivariable-adjusted HRs across the levels of MELD or ALBI scores also tended to increase for follow-up mortality (Table 4 and Supplementary Tables 5–16).

**Table 4:** Subgroup analysis of follow-up mortality based on different liver function assessment scores ^*^.

	**Multivariate analysis**			
	**MELD**		**ALBI**	
	**HR (95% CI)**	***P***	**HR (95% CI)**	***P***
**Age groups, yrs**
<65	1.05 (1.02–1.08)	<0.001	5.75 (2.32–14.27)	<0.001
≥65	1.13 (1.03–1.24)	0.008	2.46 (0.78–7.78)	0.125
**Phase of disease**
Acute	1.05 (1.02–1.08)	<0.001	2.53 (1.16–5.51)	0.019
Subacute	1.15 (1.04–1.28)	0.005	4.17 (1.37–12.73)	0.012
**Severity of disease**
Uncomplicated	1.11 (1.05–1.17)	<0.001	3.63 (1.22–10.75)	0.020
Complicated	1.05 (1.02–1.08)	0.001	4.72 (1.82–12.25)	0.001
**eGFR <60 mL/min/1.73 m**^**2**^
No	1.26 (1.12–1.43)	<0.001	3.35 (1.48–7.56)	0.004
Yes	1.04 (1.002–1.08)	0.039	1.70 (0.59–4.90)	0.324
**CAD**
No	1.05 (1.03–1.08)	<0.001	2.93 (1.43–6.02)	0.003
Yes	1.18 (1.08–1.29)	<0.001	6.74 (1.39–32.72)	0.018
**Anemia**
No	1.09 (0.97–1.22)	0.153	8.69 (2.28–33.20)	0.002
Yes	1.07 (1.04–1.10)	<0.001	2.27 (1.01–5.10)	0.047

*MELD, model of end-stage liver disease; ALBI, albumin-bilirubin; eGFR, estimated glomerular filtration rate; CAD, coronary artery disease*.

^*^Details in the Supplementary Tables 5–16.

**Figure 3:**
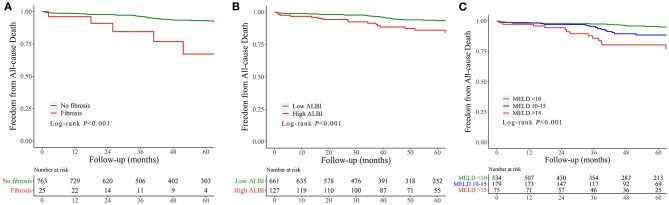
Cumulative incidence curve for follow-up mortality classified according to the fibrosis **(A)**, MELD **(B)**, and ALBI score **(C)**. MELD, model of end-stage liver disease; ALBI, albumin–bilirubin; MACE, major adverse clinical events.

Kaplan–Meier curves indicated that the cumulative follow-up mortality was significantly higher in patients with liver fibrosis (*P* < 0.001; Figure 3A), advanced MELD (*P* < 0.001, Figure 3B), or ALBI > −1.58 (*P* < 0.001, Figure 3C).

## Discussion

In the present study, we found a significant increase in in-hospital death and MACE as well as in follow-up mortality among patients with liver fibrosis and decreased liver functional reserve among TBAD patients undergoing TEVAR. Furthermore, it was demonstrated that liver fibrosis, MELD, and ALBI scores were independently associated with in-hospital death, MACE, and follow-up mortality. These data suggest that APRI, MELD, and ALBI should be calculated and incorporated into routine preoperative evaluation.

Few studies have addressed the effect of poor liver function on outcomes in TBAD patients undergoing endovascular treatment. Some researchers supported endovascular treatment for patients with poor liver function (4, 6), whereas others argued against this approach due to the risk of fatal coagulation disorder (5, 15). Temporary coagulopathy could develop after TEVAR (16). An endograft may result in endothelial damage (17), affecting coagulation activation. The liver synthesizes clotting factors and fibrinolysis-related proteins and plays a vital role in regulating the coagulation–fibrinolytic system. Consequently, patients with poor liver function are more likely to suffer from bleeding and thrombosis due to an imbalance between coagulation and fibrinolysis. Moreover, liver dysfunction might lead to the dysregulation of the immune–inflammatory system such as ongoing increasing C-reactive protein and IL-6, which originate from the liver after stimulation with cytokines and relate to the severity of dissection (18).

In a study enrolling 66,943 patients who underwent endovascular aneurysm repair, chronic liver disease was demonstrated to be a significant risk factor for 30-day mortality (OR, 2.52; *P* < 0.001) (19). Nevertheless, Chou et al. found that short-term results after endovascular therapy were promising in patients with liver cirrhosis (4). In their view, it was acceptable to perform endovascular procedures in patients with cirrhosis after careful preoperative preparation and high-quality management. In the present study, patients with poor liver function had significantly more unfavorable in-hospital outcomes than those without, even after successful TEVAR, and showed a stepwise increase with the elevation of MELD or ALBI grade. Thirteen of 24 patients deceased during hospitalization because of aortic rupture, and six patients died of multiple organ dysfunction syndrome associated with adult respiratory distress syndrome and disseminated intravascular coagulation, which implied that the importance of liver dysfunction might be underestimated in TBAD patients. Prompt intervention might be inevitable for TBAD patients when complications arise, requiring cautious perioperative preparations, including availability of blood products; individualized preoperative, intraoperative, and postoperative management; as well as advanced technology and skilled surgeons. In patients with significant liver fibrosis (OR, 23.73; *P* < 0.001), the risk of death is very high, and urgent treatment should be carried out to improve liver function and prevent fatal thromboembolic or bleeding event before the endovascular stent-graft implantation.

Consistent with previous studies (4), patients with decreased liver function experienced significantly higher follow-up mortality than those without. The result remained the same in all subgroups considered and after careful statistical adjustments. Prior studies recommended to treat advanced liver disease before cardiac surgery based on the lower survival rate in patients with advanced liver disease after cardiac surgery than those with early liver disease (20). This strategy was optimal in uncomplicated TBAD patients, whereas strict preoperative preparation and elaborate postoperative care might be preferable for complicated patients. Even after successful TEVAR, tight monitoring, timely identification, and appropriate therapy of decreased liver function are crucial to improve prognosis in those patients.

## Limitations

There are several limitations that deserve to be noted. First, we conducted a retrospective study in a single center, although we enrolled all TBAD patients undergoing TEVAR conservatively to minimize the selection bias. Second, the relatively small number of liver fibrosis patients might overestimate the percentage of morbidity and mortality, thus compromising the conclusion. Third, all included patients were Asians, and these results may not apply to other populations (e.g., a Caucasians population). Therefore, further multicenter, large prospective studies are needed to verify our findings. Fourth, due to the inclusion of patients undergoing TEVAR only, our conclusions might not be applicable to patients with conservative treatment. Lastly, the detailed mechanisms underlying why liver function impacts survival outcomes were not validated in the present study. In the future, we will perform a basic research to investigate the possible mechanisms using animal models and *in vitro* cell experiments.

## Conclusion

Liver fibrosis and decreased liver functional reserve may profoundly affect the in-hospital and follow-up outcomes. Assessment of liver function using APRI, MELD, and ALBI could provide valuable information on risk stratification and would be useful for decision-making in patients with TBAD before TEVAR.

## Data Availability Statement

The original contributions presented in the study are included in the article/Supplementary Material, further inquiries can be directed to the corresponding author/s.

## Ethics Statement

The studies involving human participants were reviewed and approved by Guangdong Provincial People's Hospital. Written informed consent for participation was not required for this study in accordance with the national legislation and the institutional requirements.

## Author Contributions

JLu, FY, and JLi were involved in the conception and the design of the study. JLi, EX, and MW collected, analyzed the data, and wrote the paper. JLi, LC, and SS performed the statistical work. HZ and QG contributed to critical revision of the manuscript for important intellectual content. All authors reviewed the manuscript and agreed with the final version.

## Conflict of Interest

The authors declare that the research was conducted in the absence of any commercial or financial relationships that could be construed as a potential conflict of interest.
