# Supplementary material for: Assessment of Liver Function for Evaluation of Short- and Long-Term Outcomes in Type B Aortic Dissection Patients Undergoing Thoracic Endovascular Aortic Repair
Source: Front Cardiovasc Med. 2021 May 12;8:643127. doi: 10.3389/fcvm.2021.643127 (PMC8190657; doi:10.3389/fcvm.2021.643127)
Supplement: Supplementary file 1 [file Data_Sheet_1.docx]

**Supplementary table 1. Cause of in-hospital death**

| **Cause of death** | **N** |
| --- | --- |
| Aortic rupture | 13 |
| MODS, ARDS and DIC | 6 |
| Organ malperfusion | 3 |
| Cardiac arrest | 1 |
| Airway compression caused by huge aortic dissecting aneurysm | 1 |

MODS, multiple organ dysfunction syndrome; ARDS, adult respiratory distress syndrome; DIC, disseminated intravascular coagulation.

**Supplementary Table 2. Details of Univariate and Multivariate Logistic regression analysis for in-hospital mortality**.

|  | Univariate | |  | Multivariate | | | | | | | |
| --- | --- | --- | --- | --- | --- | --- | --- | --- | --- | --- | --- |
|  | OR | *P* |  | APRI (>0.5 vs ≤0.5) | |  | MELD | |  | ALBI | |
|  |  |  |  | OR (95% CI) | *P* |  | OR (95% CI) | *P* |  | OR (95% CI) | *P* |
| APRI (>0.5 vs ≤0.5) | 21.8 | <0.001 |  | 23.73 (8.89-63.33) | **<0.001** |  |  |  |  |  |  |
| MELD | 1.08 | 0.006 |  |  |  |  | 1.08 (1.03-1.14) | **0.003** |  |  |  |
| ALBI | 3.43 | 0.017 |  |  |  |  |  |  |  | 4.45 (1.56-12.67) | **0.005** |
| Age | 1.03 | 0.122 |  | 1.05 (1.001-1.10) | 0.044 |  | 1.04 (1.00-1.08) | 0.064 |  |  |  |
| BMI | 1.02 | 0.740 |  |  |  |  |  |  |  |  |  |
| Complicated | 0.88 | 0.760 |  |  |  |  |  |  |  |  |  |
| CAD | 1.91 | 0.179 |  |  |  |  |  |  |  |  |  |
| Anemia | 2.18 | 0.076 |  |  |  |  |  |  |  |  |  |
| Stroke | 1.33 | 0.786 |  |  |  |  |  |  |  |  |  |
| Hypoalbuminemia | 2.39 | 0.115 |  |  |  |  |  |  |  |  |  |
| eGFR<60 mL/min/1.73 m^2^ | 2.02 | 0.096 |  |  |  |  |  |  |  |  |  |
| Creatinine, mg/dL | 1.29 | 0.012 |  |  |  |  |  |  |  |  |  |
| lg (D-dimer) | 2.50 | 0.030 |  |  |  |  |  |  |  |  |  |
| Diameter, mm | 1.06 | <0.001 |  | 1.06 (1.03-1.10) | <0.001 |  | 1.06 (1.03-1.10) | <0.001 |  | 1.07 (1.03-1.10) | <0.001 |
| Extent of the dissection | 1.50 | 0.463 |  |  |  |  |  |  |  |  |  |
| The involvement of Coeliac artery | 2.08 | 0.079 |  |  |  |  |  |  |  |  |  |
| The involvement of SMA | 3.39 | 0.004 |  | 3.37 (1.31-8.63) | 0.012 |  | 3.51 (1.47-8.37) | 0.005 |  | 3.80 (1.61-9.00) | 0.002 |

APRI, Aspartate transaminase to platelet ratio index; MELD, model of end-stage liver disease; ALBI, albumin-bilirubin; APAR, albumin-to-alkaline phosphatase ratio; BMI, body mass index; CAD, coronary artery disease; eGFR, estimated glomerular filtration rate; SMA, superior mesenteric artery.

**Supplementary Table 3. Details of Univariate and Multivariate Logistic regression analysis for in-hospital MACE**.

|  | Univariate | |  | Multivariate | | | | | | | |
| --- | --- | --- | --- | --- | --- | --- | --- | --- | --- | --- | --- |
|  | OR | *P* |  | APRI (>0.5 vs ≤0.5) | |  | MELD | |  | ALBI | |
|  |  |  |  | OR (95% CI) | *P* |  | OR (95% CI) | *P* |  | OR (95% CI) | *P* |
| APRI (>0.5 vs ≤0.5) | 7.83 | <0.001 |  | 5.92 (2.75-12.77) | **<0.001** |  |  |  |  |  |  |
| MELD | 1.09 | <0.001 |  |  |  |  | 1.05 (1.002-1.11) | **0.038** |  |  |  |
| ALBI | 2.50 | 0.003 |  |  |  |  |  |  |  | 2.22 (1.19 -4.15) | **0.013** |
| Age | 1.02 | 0.132 |  |  |  |  |  |  |  |  |  |
| BMI | 0.99 | 0.891 |  |  |  |  |  |  |  |  |  |
| Stage | 0.57 | 0.122 |  |  |  |  |  |  |  |  |  |
| Complicated | 0.99 | 0.99 |  |  |  |  |  |  |  |  |  |
| CAD | 1.75 | 0.059 |  |  |  |  |  |  |  |  |  |
| Anemia | 1.68 | 0.036 |  |  |  |  |  |  |  |  |  |
| Stroke | 1.25 | 0.358 |  | 2.47 (0.93-6.58) | 0.070 |  | 2.66 (1.03-6.86) | 0.044 |  | 2.69 (1.04-6.98) | 0.041 |
| Hypoalbuminemia | 1.81 | 0.042 |  |  |  |  |  |  |  |  |  |
| eGFR<60 mL/min/1.73 m^2^ | 2.86 | <0.001 |  | 2.29 (1.37-3.83) | 0.001 |  | 1.84 (0.97-3.47) | 0.061 |  | 2.26 (1.36-3.77) | 0.002 |
| Creatinine, mg/dL | 1.25 | 0.005 |  |  |  |  |  |  |  |  |  |
| lg (D-dimer) | 1.38 | 0.162 |  |  |  |  |  |  |  |  |  |
| Diameter, mm | 1.05 | <0.001 |  | 1.04 (1.02-1.07) | 0.001 |  | 1.04 (1.02-1.07) | <0.001 |  | 1.05 (1.02-1.07) | <0.001 |
| Extent of the dissection | 1.33 | 0.360 |  |  |  |  |  |  |  |  |  |
| The involvement of Coeliac artery | 1.39 | 0.192 |  |  |  |  |  |  |  |  |  |
| The involvement of SMA | 1.75 | 0.045 |  |  |  |  |  |  |  | 1.68 (0.95-2.96) | 0.074 |

MACE, major adverse clinical events; APRI, Aspartate transaminase to platelet ratio index; MELD, model of end-stage liver disease; ALBI, albumin-bilirubin; APAR, albumin-to-alkaline phosphatase ratio; BMI, body mass index; CAD, coronary artery disease; eGFR, estimated glomerular filtration rate; SMA, superior mesenteric artery.

**Supplementary Table 4. Details of Univariate and Multivariate Cox regression analysis for follow-up mortality**.

|  | Univariate | |  | Multivariate | | | | | | | |
| --- | --- | --- | --- | --- | --- | --- | --- | --- | --- | --- | --- |
|  | HR | *P* |  | APRI (>0.5 vs ≤0.5) | |  | MELD | |  | ALBI | |
|  |  |  |  | HR (95% CI) | *P* |  | HR (95% CI) | *P* |  | HR (95% CI) | *P* |
| APRI (>0.5 vs ≤0.5) | 4.86 | <0.001 |  | 4.69 (1.93-11.42) | **0.001** |  |  |  |  |  |  |
| MELD | 1.05 | <0.001 |  |  |  |  | 1.07 (1.04-1.10) | **<0.001** |  |  |  |
| ALBI | 3.22 | <0.001 |  |  |  |  |  |  |  | 2.88 (1.53-5.43) | **0.001** |
| Age | 1.03 | 0.003 |  | 1.02 (>0.99-1.05) | 0.064 |  | 1.03 (1.002-1.05) | 0.031 |  |  |  |
| BMI | 0.90 | 0.005 |  | 0.91 (0.84-0.99) | 0.022 |  | 0.90 (0.83-0.98) | 0.013 |  | 0.92 (0.85-0.99) | 0.028 |
| Stage | 1.56 | 0.102 |  |  |  |  | 1.64 (0.95-2.81) | 0.074 |  |  |  |
| Complicated | 0.92 | 0.753 |  |  |  |  |  |  |  |  |  |
| CAD | 2.09 | 0.006 |  | 1.72 (0.98-3.01) | 0.057 |  | 1.70 (0.97-2.99) | 0.064 |  | 1.99 (1.17-3.39) | 0.011 |
| Anemia | 1.98 | 0.007 |  |  |  |  |  |  |  |  |  |
| Stroke | 2.10 | 0.211 |  |  |  |  |  |  |  |  |  |
| Hypoalbuminemia | 1.78 | 0.071 |  |  |  |  |  |  |  |  |  |
| eGFR<60 mL/min/1.73 m^2^ | 2.17 | 0.001 |  | 1.85 (1.13-3.04) | 0.014 |  |  |  |  | 1.84 (1.13-3.01) | 0.015 |
| Creatinine, mg/dL | 1.21 | 0.010 |  |  |  |  |  |  |  |  |  |
| lg (D-dimer) | 1.35 | 0.196 |  |  |  |  |  |  |  |  |  |
| Diameter, mm | 1.01 | 0.587 |  |  |  |  |  |  |  |  |  |
| Extent of the dissection | 1.44 | 0.223 |  |  |  |  |  |  |  |  |  |
| The involvement of Coeliac artery | 1.002 | 0.994 |  |  |  |  |  |  |  |  |  |
| The involvement of SMA | 1.26 | 0.468 |  |  |  |  |  |  |  |  |  |

APRI, Aspartate transaminase to platelet ratio index; MELD, model of end-stage liver disease; ALBI, albumin-bilirubin; APAR, albumin-to-alkaline phosphatase ratio; BMI, body mass index; CAD, coronary artery disease; eGFR, estimated glomerular filtration rate; SMA, superior mesenteric artery.

**Supplementary Table 5. Details of Cox regression analysis for follow-up mortality in patients with age ≥65 years**

|  | Univariate | |  | Multivariate | | | | |
| --- | --- | --- | --- | --- | --- | --- | --- | --- |
|  | HR | *P* |  | MELD | |  | ALBI | |
|  |  |  |  | HR (95% CI) | *P* |  | HR (95% CI) | *P* |
| MELD | 1.15 | 0.002 |  | 1.13 (1.03-1.24) | **0.008** |  |  |  |
| ALBI | 2.20 | 0.154 |  |  |  |  | 2.46 (0.78-7.78) | **0.125** |
| BMI | 0.96 | 0.521 |  |  |  |  | 0.86 (0.74-0.99) | 0.037 |
| Complicated | 0.52 | 0.126 |  |  |  |  | 0.45 (0.20-1.05) | 0.064 |
| CAD | 2.46 | 0.032 |  | 2.49 (1.08-5.76) | 0.032 |  | 4.22 (1.83-9.74) | 0.001 |
| Anemia | 1.53 | 0.351 |  |  |  |  |  |  |
| Stroke | 1.58 | 0.654 |  |  |  |  |  |  |
| Hypoalbuminemia | 3.71 | 0.081 |  | 3.39 (0.75-15.35) | 0.114 |  |  |  |
| eGFR<60 mL/min/1.73 m^2^ | 3.16 | 0.009 |  |  |  |  | 3.39 (1.42-8.09) | 0.006 |
| Creatinine, mg/dL | 1.36 | 0.042 |  |  |  |  |  |  |
| lg (D-dimer) | 1.94 | 0.108 |  |  |  |  |  |  |
| Diameter, mm | 1.002 | 0.943 |  |  |  |  |  |  |
| Extent of the dissection | 1.95 | 0.188 |  |  |  |  |  |  |
| The involvement of Coeliac artery | 1.61 | 0.357 |  |  |  |  |  |  |
| The involvement of SMA | 0.90 | 0.882 |  |  |  |  |  |  |

MELD, model of end-stage liver disease; ALBI, albumin-bilirubin; BMI, body mass index; CAD, coronary artery disease; eGFR, estimated glomerular filtration rate; SMA, superior mesenteric artery.

**Supplementary Table 6. Details of Cox regression analysis for follow-up mortality in patients with age <65 years**

|  | Univariate | |  | Multivariate | | | | |
| --- | --- | --- | --- | --- | --- | --- | --- | --- |
|  | HR | *P* |  | MELD | |  | ALBI | |
|  |  |  |  | HR (95% CI) | *P* |  | HR (95% CI) | *P* |
| MELD | 1.05 | <0.001 |  | 1.05 (1.02-1.08) | **<0.001** |  |  |  |
| ALBI | 3.64 | 0.001 |  |  |  |  | 5.75 (2.32-14.27) | **<0.001** |
| BMI | 0.89 | 0.013 |  | 0.88 (0.80-0.97) | 0.010 |  | 0.88 (0.80-0.97) | 0.013 |
| Complicated | 1.38 | 0.337 |  |  |  |  |  |  |
| CAD | 1.59 | 0.213 |  |  |  |  |  |  |
| Anemia | 2.11 | 0.016 |  | 1.72 (0.92-3.21) | 0.087 |  |  |  |
| Stroke | 2.29 | 0.254 |  |  |  |  |  |  |
| Hypoalbuminemia | 1.41 | 0.342 |  |  |  |  | 0.44 (0.18-1.08) | 0.072 |
| eGFR<60 mL/min/1.73 m^2^ | 1.76 | 0.069 |  |  |  |  |  |  |
| Creatinine, mg/dL | 1.21 | 0.034 |  |  |  |  |  |  |
| lg (D-dimer) | 1.16 | 0.698 |  |  |  |  |  |  |
| Diameter, mm | 1.009 | 0.601 |  |  |  |  |  |  |
| Extent of the dissection | 1.32 | 0.451 |  |  |  |  |  |  |
| The involvement of Coeliac artery | 0.95 | 0.878 |  |  |  |  |  |  |
| The involvement of SMA | 1.48 | 0.277 |  |  |  |  |  |  |

MELD, model of end-stage liver disease; ALBI, albumin-bilirubin; BMI, body mass index; CAD, coronary artery disease; eGFR, estimated glomerular filtration rate; SMA, superior mesenteric artery.

**Supplementary Table 7. Details of Cox regression analysis for follow-up mortality in patients with acute aortic dissection**

|  | Univariate | |  | | Multivariate | | | | |
| --- | --- | --- | --- | --- | --- | --- | --- | --- | --- |
|  | HR | *P* |  | MELD | | |  | ALBI | |
|  |  |  |  | HR (95% CI) | | *P* |  | HR (95% CI) | *P* |
| MELD | 1.05 | <0.001 |  | 1.05 (1.02-1.08) | | **<0.001** |  |  |  |
| ALBI | 2.79 | 0.008 |  |  | |  |  | 2.53 (1.16-5.52) | **0.019** |
| Age | 1.02 | 0.157 |  |  | |  |  |  |  |
| BMI | 0.91 | 0.030 |  | 0.91 (0.83-0.997) | | 0.042 |  | 0.92 (0.84-1.00) | 0.055 |
| Complicated | 1.47 | 0.275 |  |  | |  |  |  |  |
| CAD | 2.12 | 0.020 |  | 2.00 (1.05-3.81) | | 0.035 |  | 1.85 (0.98-3.51) | 0.057 |
| Anemia | 2.17 | 0.010 |  | 1.73 (0.94-3.17) | | 0.077 |  |  |  |
| Stroke | 1.53 | 0.676 |  |  | |  |  |  |  |
| Hypoalbuminemia | 1.28 | 0.483 |  |  | |  |  |  |  |
| eGFR<60 mL/min/1.73 m^2^ | 2.15 | 0.007 |  |  | |  |  | 1.90 (1.07-3.35) | 0.027 |
| Creatinine, mg/dL | 1.19 | 0.041 |  |  | |  |  |  |  |
| lg (D-dimer) | 1.07 | 0.806 |  |  | |  |  |  |  |
| Diameter, mm | 1.02 | 0.156 |  |  | |  |  |  |  |
| Extent of the dissection | 1.47 | 0.298 |  |  | |  |  |  |  |
| The involvement of Coeliac artery | 1.11 | 0.741 |  |  | |  |  |  |  |
| The involvement of SMA | 1.36 | 0.391 |  |  | |  |  |  |  |

MELD, model of end-stage liver disease; ALBI, albumin-bilirubin; BMI, body mass index; CAD, coronary artery disease; eGFR, estimated glomerular filtration rate; SMA, superior mesenteric artery.

**Supplementary Table 8. Details of Cox regression analysis for follow-up mortality in patients with subacute aortic dissection**

|  | Univariate | |  | Multivariate | | | | |
| --- | --- | --- | --- | --- | --- | --- | --- | --- |
|  | HR | *P* |  | MELD | |  | ALBI | |
|  |  |  |  | HR (95% CI) | *P* |  | HR (95% CI) | *P* |
| MELD | 1.18 | 0.001 |  | 1.15 (1.04-1.28) | **0.005** |  |  |  |
| ALBI | 4.74 | 0.002 |  |  |  |  | 4.17 (1.37-12.73) | **0.012** |
| Age | 1.08 | 0.001 |  | 1.06 (1.02-1.11) | 0.007 |  | 1.08 (1.03-1.13) | 0.001 |
| BMI | 0.88 | 0.085 |  |  |  |  |  |  |
| Complicated | 0.50 | 0.232 |  |  |  |  |  |  |
| CAD | 1.78 | 0.245 |  |  |  |  |  |  |
| Anemia | 1.39 | 0.508 |  |  |  |  |  |  |
| Stroke | 2.21 | 0.295 |  |  |  |  |  |  |
| Hypoalbuminemia | 5.80 | 0.019 |  | 3.39 (0.76-15.18) | 0.111 |  |  |  |
| eGFR<60 mL/min/1.73 m^2^ | 2.56 | 0.049 |  |  |  |  |  |  |
| Creatinine, mg/dL | 3.14 | 0.003 |  |  |  |  | 2.49 (1.09-5.69) | 0.031 |
| lg (D-dimer) | 2.74 | 0.022 |  |  |  |  |  |  |
| Diameter, mm | 0.97 | 0.233 |  |  |  |  |  |  |
| Extent of the dissection | 1.69 | 0.318 |  |  |  |  |  |  |
| The involvement of Coeliac artery | 0.78 | 0.698 |  |  |  |  |  |  |
| The involvement of SMA | 1.08 | 0.914 |  |  |  |  |  |  |

MELD, model of end-stage liver disease; ALBI, albumin-bilirubin; BMI, body mass index; CAD, coronary artery disease; eGFR, estimated glomerular filtration rate; SMA, superior mesenteric artery.

**Supplementary Table 9. Details of Cox regression analysis for follow-up mortality in patients with complicated aortic dissection**

|  | Univariate | |  | Multivariate | | | | |
| --- | --- | --- | --- | --- | --- | --- | --- | --- |
|  | HR | *P* |  | MELD | |  | ALBI | |
|  |  |  |  | HR (95% CI) | *P* |  | HR (95% CI) | *P* |
| MELD | 1.04 | 0.004 |  | 1.05 (1.02-1.08) | **0.001** |  |  |  |
| ALBI | 2.99 | 0.006 |  |  |  |  | 4.72 (1.82-12.25) | **0.001** |
| Age | 1.02 | 0.169 |  |  |  |  |  |  |
| BMI | 0.89 | 0.014 |  | 0.87 (0.79-0.96) | 0.004 |  | 0.88 (0.80-0.97) | 0.010 |
| CAD | 1.55 | 0.243 |  |  |  |  |  |  |
| Anemia | 1.68 | 0.101 |  |  |  |  |  |  |
| Stroke | 1.60 | 0.645 |  |  |  |  |  |  |
| Hypoalbuminemia | 1.21 | 0.597 |  |  |  |  | 0.44 (0.18-1.07) | 0.069 |
| eGFR<60 mL/min/1.73 m^2^ | 2.21 | 0.009 |  |  |  |  | 1.81 (0.98-3.32) | 0.057 |
| Creatinine, mg/dL | 1.26 | 0.020 |  |  |  |  |  |  |
| lg (D-dimer) | 1.06 | 0.853 |  |  |  |  |  |  |
| Diameter, mm | 1.03 | 0.071 |  | 1.04 (1.004-1.07) | 0.027 |  | 1.04 (1.005-1.08) | 0.024 |
| Extent of the dissection | 1.27 | 0.519 |  |  |  |  |  |  |
| The involvement of Coeliac artery | 0.92 | 0.815 |  |  |  |  |  |  |
| The involvement of SMA | 1.22 | 0.613 |  |  |  |  |  |  |

MELD, model of end-stage liver disease; ALBI, albumin-bilirubin; BMI, body mass index; CAD, coronary artery disease; eGFR, estimated glomerular filtration rate; SMA, superior mesenteric artery.

**Supplementary Table 10. Details of Cox regression analysis for follow-up mortality in patients with uncomplicated aortic dissection**

|  | Univariate | |  | Multivariate | | | | |
| --- | --- | --- | --- | --- | --- | --- | --- | --- |
|  | HR | *P* |  | MELD | |  | ALBI | |
|  |  |  |  | HR (95% CI) | *P* |  | HR (95% CI) | *P* |
| MELD | 1.09 | 0.003 |  | 1.11 (1.05-1.17) | **<0.001** |  |  |  |
| ALBI | 3.35 | 0.015 |  |  |  |  | 3.63 (1.22-10.75) | **0.020** |
| Age | 1.06 | 0.002 |  | 1.05 (1.01-1.09) | 0.020 |  | 1.04 (0.99-1.09) | 0.058 |
| BMI | 0.91 | 0.162 |  |  |  |  |  |  |
| CAD | 3.24 | 0.005 |  | 2.24 (0.91-5.52) | 0.078 |  | 2.52 (1.03-6.20) | 0.043 |
| Anemia | 2.84 | 0.022 |  |  |  |  |  |  |
| Stroke | 2.45 | 0.229 |  |  |  |  |  |  |
| Hypoalbuminemia | 4.64 | 0.038 |  |  |  |  |  |  |
| eGFR<60 mL/min/1.73 m^2^ | 2.08 | 0.073 |  |  |  |  |  |  |
| Creatinine, mg/dL | 1.17 | 0.185 |  |  |  |  |  |  |
| lg (D-dimer) | 1.99 | 0.068 |  |  |  |  |  |  |
| Diameter, mm | 0.98 | 0.333 |  |  |  |  |  |  |
| Extent of the dissection | 1.82 | 0.238 |  |  |  |  |  |  |
| The involvement of Coeliac artery | 1.18 | 0.722 |  |  |  |  |  |  |
| The involvement of SMA | 1.36 | 0.578 |  |  |  |  |  |  |

MELD, model of end-stage liver disease; ALBI, albumin-bilirubin; BMI, body mass index; CAD, coronary artery disease; eGFR, estimated glomerular filtration rate; SMA, superior mesenteric artery.

**Supplementary Table 11. Details of Cox regression analysis for follow-up mortality in patients with eGFR<60 mL/min/1.73 m^2^**

|  | Univariate | |  | | Multivariate | | | | |
| --- | --- | --- | --- | --- | --- | --- | --- | --- | --- |
|  | HR | *P* |  | MELD | | |  | ALBI | |
|  |  |  |  | HR (95% CI) | | *P* |  | HR (95% CI) | *P* |
| MELD | 1.03 | 0.092 |  | 1.04 (1.002-1.08) | | **0.039** |  |  |  |
| ALBI | 2.25 | 0.110 |  |  | |  |  | 1.70 (0.59-4.90) | 0.324 |
| Age | 1.04 | 0.022 |  | 1.05 (1.01-1.08) | | 0.013 |  | 1.04 (1.01-1.08) | 0.021 |
| BMI | 0.90 | 0.097 |  |  | |  |  |  |  |
| Stage | 1.93 | 0.132 |  |  | |  |  |  |  |
| Complicated | 0.96 | 0.91 |  |  | |  |  |  |  |
| CAD | 1.55 | 0.294 |  |  | |  |  |  |  |
| Anemia | 3.36 | 0.014 |  | 2.75 (1.01-7.45) | | 0.047 |  | 2.65 (0.96-7.29) | 0.060 |
| Stroke | 2.10 | 0.318 |  |  | |  |  |  |  |
| Hypoalbuminemia | 2.11 | 0.223 |  |  | |  |  |  |  |
| lg (D-dimer) | 1.17 | 0.672 |  |  | |  |  |  |  |
| Diameter, mm | 1.008 | 0.749 |  |  | |  |  |  |  |
| Extent of the dissection | 1.47 | 0.435 |  |  | |  |  | 1.60 (0.58-4.41) | 0.363 |
| The involvement of Coeliac artery | 0.84 | 0.698 |  |  | |  |  |  |  |
| The involvement of SMA | 1.43 | 0.394 |  |  | |  |  | 1.51 (0.63-3.63) | 0.355 |

MELD, model of end-stage liver disease; ALBI, albumin-bilirubin; BMI, body mass index; CAD, coronary artery disease; eGFR, estimated glomerular filtration rate; SMA, superior mesenteric artery.

**Supplementary Table 12. Details of Cox regression analysis for follow-up mortality in patients with eGFR≥60 mL/min/1.73 m^2^**

|  | Univariate | |  | | Multivariate | | | | |
| --- | --- | --- | --- | --- | --- | --- | --- | --- | --- |
|  | HR | *P* |  | MELD | | |  | ALBI | |
|  |  |  |  | HR (95% CI) | | *P* |  | HR (95% CI) | *P* |
| MELD | 1.25 | <0.001 |  | 1.26 (1.12-1.43) | | **<0.001** |  |  |  |
| ALBI | 3.08 | 0.005 |  |  | |  |  | 3.35 (1.48-7.56) | **0.004** |
| Age | 1.03 | 0.100 |  |  | |  |  |  |  |
| BMI | 0.89 | 0.020 |  | 0.91 (0.82-1.004) | | 0.059 |  | 0.92 (0.83-1.01) | 0.092 |
| Stage | 1.56 | 0.198 |  | 1.90 (0.94-3.87) | | 0.075 |  | 1.88 (0.93-3.78) | 0.079 |
| Complicated | 0.90 | 0.755 |  |  | |  |  |  |  |
| CAD | 2.20 | 0.026 |  | 2.21 (1.09-4.44) | | 0.027 |  | 2.12 (1.06-4.26) | 0.034 |
| Anemia | 1.37 | 0.324 |  |  | |  |  |  |  |
| Stroke | 1.71 | 0.597 |  |  | |  |  |  |  |
| Hypoalbuminemia | 1.41 | 0.366 |  |  | |  |  |  |  |
| lg (D-dimer) | 1.33 | 0.336 |  |  | |  |  |  |  |
| Diameter, mm | 1.003 | 0.870 |  |  | |  |  |  |  |
| Extent of the dissection | 1.40 | 0.377 |  |  | |  |  |  |  |
| The involvement of Coeliac artery | 1.11 | 0.771 |  |  | |  |  |  |  |
| The involvement of SMA | 0.77 | 0.622 |  |  | |  |  |  |  |

MELD, model of end-stage liver disease; ALBI, albumin-bilirubin; BMI, body mass index; CAD, coronary artery disease; eGFR, estimated glomerular filtration rate; SMA, superior mesenteric artery.

**Supplementary Table 13. Details of Cox regression analysis for follow-up mortality in patients with coronary artery disease**

|  | Univariate | |  | | Multivariate | | | | |
| --- | --- | --- | --- | --- | --- | --- | --- | --- | --- |
|  | HR | *P* |  | MELD | | |  | ALBI | |
|  |  |  |  | HR (95% CI) | | *P* |  | HR (95% CI) | *P* |
| MELD | 1.16 | <0.001 |  | 1.18 (1.08-1.29) | | **<0.001** |  |  |  |
| ALBI | 4.12 | 0.029 |  |  | |  |  | 6.74 (1.39-32.72) | **0.018** |
| Age | 1.08 | 0.012 |  | 1.08 (1.01-1.14) | | 0.015 |  | 1.05 (0.99-1.11) | 0.127 |
| BMI | 0.84 | 0.043 |  | 0.84 (0.71-1.004) | | 0.055 |  | 0.85 (0.72-1.01) | 0.073 |
| Stage | 1.31 | 0.584 |  |  | |  |  | 3.26 (0.96-11.05) | 0.058 |
| Complicated | 0.50 | 0.140 |  |  | |  |  |  |  |
| Anemia | 4.62 | 0.015 |  |  | |  |  |  |  |
| Stroke | 0.05 | 0.481 |  |  | |  |  |  |  |
| Hypoalbuminemia | 3.36 | 0.107 |  |  | |  |  |  |  |
| eGFR<60 mL/min/1.73 m^2^ | 1.94 | 0.159 |  |  | |  |  |  |  |
| Creatinine, mg/dL | 1.39 | 0.040 |  |  | |  |  | 1.63 (1.09-2.42) | 0.017 |
| lg (D-dimer) | 1.72 | 0.203 |  |  | |  |  |  |  |
| Diameter, mm | 0.99 | 0.789 |  |  | |  |  |  |  |
| Extent of the dissection | 0.99 | 0.976 |  |  | |  |  |  |  |
| The involvement of Coeliac artery | 0.67 | 0.525 |  |  | |  |  |  |  |
| The involvement of SMA | 1.09 | 0.897 |  |  | |  |  |  |  |

MELD, model of end-stage liver disease; ALBI, albumin-bilirubin; BMI, body mass index; eGFR, estimated glomerular filtration rate; SMA, superior mesenteric artery.

**Supplementary Table 14. Details of Cox regression analysis for follow-up mortality in patients without coronary artery disease**

|  | Univariate | |  | | Multivariate | | | | |
| --- | --- | --- | --- | --- | --- | --- | --- | --- | --- |
|  | HR | *P* |  | MELD | | |  | ALBI | |
|  |  |  |  | HR (95% CI) | | *P* |  | HR (95% CI) | *P* |
| MELD | 1.05 | <0.001 |  | 1.05 (1.03-1.08) | | **<0.001** |  |  |  |
| ALBI | 3.18 | 0.001 |  |  | |  |  | 2.93 (1.43-6.02) | **0.003** |
| Age | 1.02 | 0.129 |  |  | |  |  |  |  |
| BMI | 0.92 | 0.044 |  | 0.90 (0.82-0.99) | | 0.022 |  |  |  |
| Stage | 1.55 | 0.177 |  |  | |  |  | 1.77 (0.94-3.35) | 0.079 |
| Complicated | 1.21 | 0.544 |  |  | |  |  |  |  |
| Anemia | 1.53 | 0.141 |  |  | |  |  |  |  |
| Stroke | 4.35 | 0.015 |  | 3.74 (1.14-12.23) | | 0.029 |  | 3.89 (1.18-12.83) | 0.026 |
| Hypoalbuminemia | 1.52 | 0.241 |  |  | |  |  |  |  |
| eGFR<60 mL/min/1.73 m^2^ | 2.25 | 0.005 |  |  | |  |  | 1.86 (1.04-3.33) | 0.036 |
| Creatinine, mg/dL | 1.20 | 0.035 |  |  | |  |  |  |  |
| lg (D-dimer) | 1.31 | 0.326 |  |  | |  |  |  |  |
| Diameter, mm | 1.01 | 0.545 |  |  | |  |  |  |  |
| Extent of the dissection | 1.82 | 0.122 |  |  | |  |  |  |  |
| The involvement of Coeliac artery | 1.14 | 0.673 |  |  | |  |  |  |  |
| The involvement of SMA | 1.29 | 0.492 |  |  | |  |  |  |  |

MELD, model of end-stage liver disease; ALBI, albumin-bilirubin; BMI, body mass index; eGFR, estimated glomerular filtration rate; SMA, superior mesenteric artery.

**Supplementary Table 15. Details of Cox regression analysis for follow-up mortality in patients with anemia**

|  | Univariate | |  | | Multivariate | | | | |
| --- | --- | --- | --- | --- | --- | --- | --- | --- | --- |
|  | HR | *P* |  | MELD | | |  | ALBI | |
|  |  |  |  | HR (95% CI) | | *P* |  | HR (95% CI) | *P* |
| MELD | 1.04 | <0.001 |  | 1.07 (1.04-1.10) | | **<0.001** |  |  |  |
| ALBI | 2.71 | 0.010 |  |  | |  |  | 2.27 (1.01-5.10) | **0.047** |
| Age | 1.04 | 0.007 |  | 1.03 (1.002-1.07) | | 0.038 |  |  |  |
| BMI | 0.90 | 0.020 |  | 0.90 (0.81-1.003) | | 0.057 |  | 0.91 (0.83-1.01) | 0.070 |
| Stage | 1.25 | 0.490 |  |  | |  |  |  |  |
| Complicated | 0.77 | 0.396 |  |  | |  |  |  |  |
| CAD | 2.74 | 0.001 |  | 1.98 (1.03-3.83) | | 0.041 |  | 2.42 (1.30-4.49) | 0.005 |
| Stroke | 2.06 | 0.229 |  |  | |  |  |  |  |
| Hypoalbuminemia | 3.39 | 0.041 |  |  | |  |  |  |  |
| eGFR<60 mL/min/1.73 m^2^ | 2.63 | 0.001 |  |  | |  |  | 2.31 (1.28-4.16) | 0.005 |
| Creatinine, mg/dL | 1.18 | 0.030 |  |  | |  |  |  |  |
| lg (D-dimer) | 1.50 | 0.161 |  |  | |  |  |  |  |
| Diameter, mm | 1.01 | 0.552 |  |  | |  |  |  |  |
| Extent of the dissection | 1.91 | 0.116 |  |  | |  |  |  |  |
| The involvement of Coeliac artery | 1.20 | 0.587 |  |  | |  |  |  |  |
| The involvement of SMA | 1.90 | 0.073 |  | 1.89 (0.92-3.86) | | 0.082 |  |  |  |

MELD, model of end-stage liver disease; ALBI, albumin-bilirubin; BMI, body mass index; CAD, coronary artery disease; eGFR, estimated glomerular filtration rate; SMA, superior mesenteric artery.

**Supplementary Table 16. Details of Cox regression analysis for follow-up mortality in patients without anemia**

|  | Univariate | |  | Multivariate | | | | |
| --- | --- | --- | --- | --- | --- | --- | --- | --- |
|  | HR | *P* |  | MELD | |  | ALBI | |
|  |  |  |  | HR (95% CI) | *P* |  | HR (95% CI) | *P* |
| MELD | 1.07 | 0.272 |  | 1.09 (0.97-1.22) | 0.153 |  |  |  |
| ALBI | 2.72 | 0.090 |  |  |  |  | 8.69 (2.28-33.20) | **0.002** |
| Age | 1.02 | 0.459 |  |  |  |  |  |  |
| BMI | 0.94 | 0.350 |  |  |  |  |  |  |
| Stage | 1.95 | 0.163 |  | 2.25 (0.86-5.89) | 0.097 |  | 2.91 (1.08-7.85) | 0.035 |
| Complicated | 1.28 | 0.593 |  |  |  |  |  |  |
| CAD | 0.90 | 0.870 |  |  |  |  |  |  |
| Hypoalbuminemia | 0.88 | 0.759 |  |  |  |  | 0.34 (0.11-1.02) | 0.035 |
| eGFR<60 mL/min/1.73 m^2^ | 1.11 | 0.844 |  |  |  |  |  |  |
| Creatinine, mg/dL | 1.13 | 0.722 |  |  |  |  |  |  |
| lg (D-dimer) | 1.00 | 0.999 |  |  |  |  |  |  |
| Diameter, mm | 1.004 | 0.884 |  |  |  |  |  |  |
| Extent of the dissection | 0.88 | 0.777 |  |  |  |  |  |  |
| The involvement of Coeliac artery | 0.76 | 0.596 |  |  |  |  |  |  |
| The involvement of SMA | 0.50 | 0.356 |  |  |  |  |  |  |

MELD, model of end-stage liver disease; ALBI, albumin-bilirubin; BMI, body mass index; CAD, coronary artery disease; eGFR, estimated glomerular filtration rate; SMA, superior mesenteric artery.
